# Quality and Flavor Evolution of *Pugionium cornutum* (L.) Gaertn. During Natural Pickling: Insights from Sensory and Physicochemical Characterizations

**DOI:** 10.3390/foods15193513

**Published:** 2026-10-01

**Authors:** Haoyu Li, Shuoshuo Kang, Yanrui Wu, Yuxuan Zhang, Jing Du, Rui Tian, Hao Yu

**Affiliations:** 1 Shaanxi Key Laboratory of Chemical Reaction Engineering, Engineering Technology Research Center for Food Analysis and Evaluation of Yan’an City, College of Chemistry and Chemical Engineering, Yan’an University, Yan’an 716000, China; 2 Agricultural Products Quality and Safety Inspection and Testing Center of Yan’an City, Yan’an 716000, China

**Keywords:** *P. cornutum*, natural pickling, E-nose, HS-GC-IMS, VOCs, flavor evolution, multivariate analysis

## Abstract

*Pugionium cornutum* (L.) Gaertn. is an underutilized *Brassicaceae* vegetable whose strong pungency limits its broader food-industrial application. Natural pickling can mitigate pungency and improve flavor, yet the dynamic evolution of flavor quality, physicochemical shifts, volatile profiles and sensory maturation throughout natural pickling remains poorly understood. Herein, sensory evaluation, physicochemical assays, electronic nose (E-nose), headspace gas chromatography–ion mobility spectrometry (HS-GC-IMS) and multivariate analyses were adopted to characterize its quality and flavor changes during 28-day natural pickling. The first 7 days represented the major physicochemical transformation interval, featuring rapid NaCl uptake, pH drop, increased titratable acidity (TA) and amino nitrogen (amino-N), reducing-sugar consumption, and a transient nitrite peak. Weighted sensory score increased from 42.75 (D0) to 81.84–83.98 (D21-D28). Aroma, taste, and texture were enhanced despite declining color scores, with no significant difference in overall acceptability between D21 and D28. E-nose principal component analysis (PCA) explained 91.78% total variance and revealed a D21–D28 overlap. HS-GC-IMS detected 84 signal peaks and tentatively identified 75 volatile organic compounds (VOCs). Aldehydes and sulfur-related volatiles generally decreased, whereas esters, ketones, and organic-acid-related compounds accumulated. In total, 33 differential VOCs with variable importance in projection (VIP) > 1 were discriminated during the pickling stage. Correlation analysis revealed associations among physicochemical variables, E-nose responses, and VOC patterns. Results indicate that natural pickling progressively optimizes sensory attributes, reaching a relatively stable aroma state at day 21. This study supplies references for pickling endpoints judgment and supports the processing development of this under exploited vegetable resource.

## 1. Introduction

*Pugionium cornutum* (L.) Gaertn. (hereafter referred to as *P. cornutum*) is an herbaceous plant of the genus *Pugionium* in the family *Brassicaceae*, and is widely distributed in the arid and semi-arid regions of northwestern China. *P. cornutum* exhibits strong tolerance to drought, salinity, wind erosion, and nutrient-poor sandy soils [1], which gives the plant ecological value. Its tender stems and leaves are rich in protein, dietary fiber, as well as abundant free amino acids, vitamins, minerals and bioactive substances [2,3]. Moreover, *P. cornutum* has a long history of application in traditional Mongolian medicine, where it is recognized for its effects in relieving pain, suppressing coughs, and promoting digestion [2,4].

However, as a traditional seasonal wild vegetable, *P. cornutum* suffers from a short harvest window, and its tender tissues are susceptible to water loss, senescence, and microbial deterioration. In addition, fresh *P. cornutum* possesses a strong, pungent odor that, although accepted in some traditional diets, generally limits broader consumer acceptance due to sensory concerns. The combination of a concentrated harvest period, poor storability, and distinctive flavor presents significant challenges for fresh market sales. Thus, processing is essential to extend its shelf life and improve palatability, yet systematic studies on its processing characteristics and sensory quality remain scarce.

Pickling, as a time-honored vegetable preservation technique, has been practiced worldwide for thousands of years. During pickling, microbial metabolism, endogenous enzymatic reactions, osmotic transfer, and changes in the physicochemical environment can reshape product color, texture, taste, and aroma [5,6,7]. For *P. cornutum*, whose pungent odor often reduces its appeal as a fresh vegetable, pickling serves not only to extend shelf life but also holds potential for odor moderation via fermentation or flavor masking. This could lead to a softer and more complex flavor profile, which might be better accepted by consumers who are sensitive to strong smells. Although pickling represents a traditional edible method for *P. cornutum*, existing studies mostly focus on volatile compounds of fresh and dehydrated materials [8]. To date, little information is available on volatile composition and flavor evolution during *P. cornutum* pickling. Therefore, the time-dependent evolution of volatile compounds during *P. cornutum* pickling remains unclear and requires systematic investigation.

In food flavor research, sensory evaluation directly reflects consumer acceptance, while physicochemical indices objectively characterize the maturation degree of pickled products. E-nose can rapidly capture the overall volatile spatial response patterns and enable rapid differentiation based on processing stage or sample variations, although their metal-oxide sensors exhibit cross-sensitivity rather than specificity [9,10], and HS-GC-IMS exhibits high sensitivity for the identification of trace volatile compounds [11,12]. The combined application of E-nose and HS-GC-IMS has been proven effective in elaborating the dynamic flavor evolution of fermented and pickled foods.

Therefore, this study investigate the changes in sensory, physicochemical characteristics, volatile flavor profiles, and odor fingerprints of *P. cornutum* during pickling. Multivariate statistical analyses including PCA and partial least squares discriminant analysis (PLS-DA) were applied to distinguish samples at different pickling stages and screen key differential volatile compounds. Correlation analysis was further performed to clarify the relationships between E-nose response, flavor substances, and physicochemical indices. This work aims to clarify the flavor formation mechanism of pickled *P. cornutum* and provide theoretical support for its processing optimization and quality control.

## 2. Materials and Methods

### 2.1. Preparation of P. Cornutum Pickles

**Figure 1:**
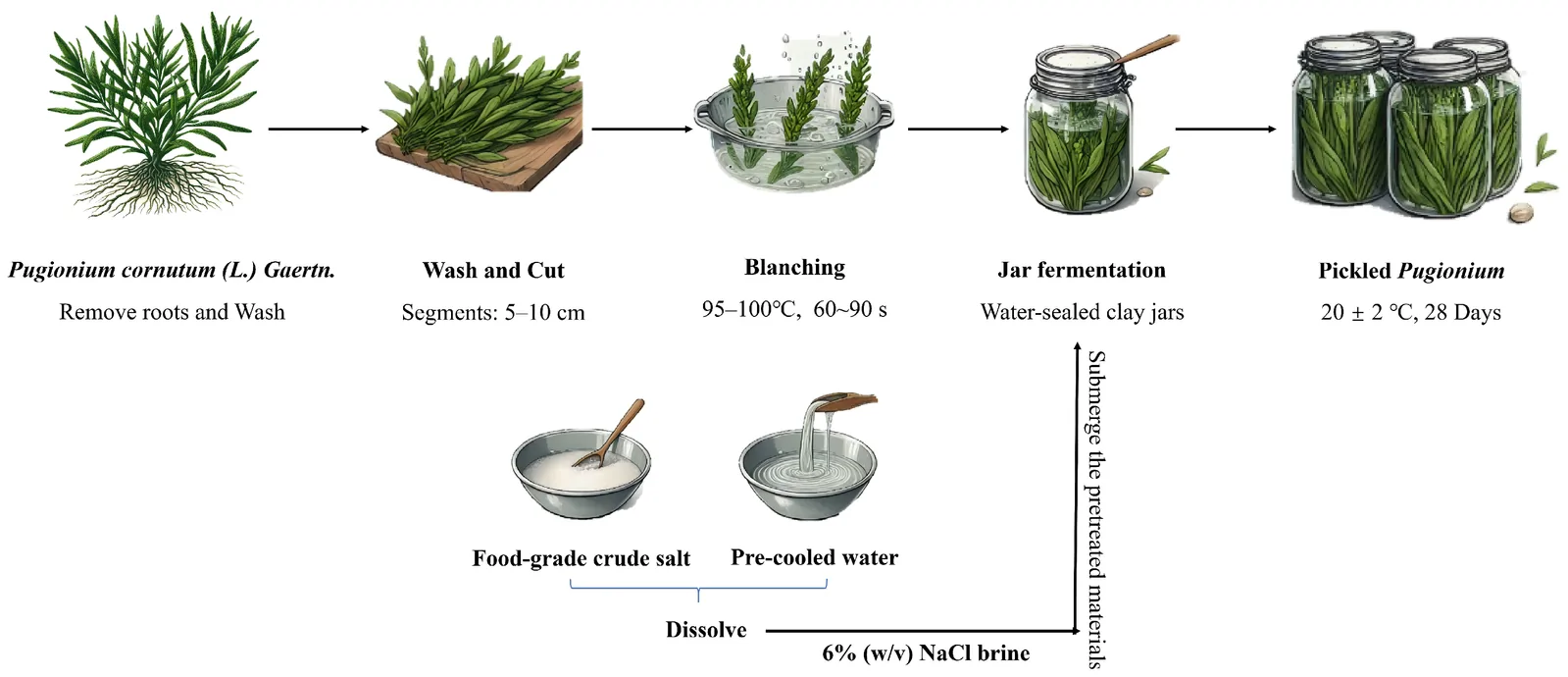
Schematic illustration of the natural pickling process of *P. cornutum*.

Fresh *P. cornutum* and food-grade crude salt were purchased from a local market in Yulin, Shaanxi Province, China. The preparation of *P. cornutum* pickles followed a traditional natural fermentation procedure (Figure 1). Briefly, fresh *P. cornutum* was washed thoroughly, cut into uniform segments (5–10 cm), blanched in boiling water (95–100 °C) for 60–90 s, and immediately cooled under running tap water. After draining excess surface moisture, the pretreated material (approximately 2.5 kg per jar) was transferred into 10 L clay jars. Pre-boiled and cooled (22–25 °C) brine solution containing 6% (*w*/*v*) NaCl was added to each jar to fully submerge the material. A 5–8 cm headspace was reserved below the jar rim for gas production during pickling, guaranteeing consistent immersion conditions across all jars. The rim was filled with water, and the jars underwent natural fermentation at 20 ± 2 °C for 28 days. Samples of about 200 g (including solids and brine) were taken from three parallel clay jars on days 0, 7, 14, 21, and 28 of pickling, and designated as D0, D7, D14, D21, and D28, respectively; no additional liquid was added after sampling. The D0 sample was collected immediately after blanching, cooling, and immersion in brine and was defined as the pretreated sample before fermentation.

All samples were immediately packaged in sterile polyethylene bags after collection. Samples for physicochemical analysis were tested immediately. For overall flavor and volatile flavor compounds analysis, the samples were rapidly frozen and stored at –80 °C. After completion of the whole experimental period, all samples were analyzed under the same analytical conditions to reduce variations introduced by batch-to-batch testing.

### 2.2. Sensory Evaluation

Samples of *P. cornutum* pickled at different pickling times were subjected to sensory evaluation immediately after sampling (without frozen storage) by eight trained panelists with experience in food sensory assessment. Before evaluation, panelists were familiarized with the typical sensory characteristics of pickled *P. cornutum* and with the definitions and scoring scales for each attribute. Samples were randomly coded and presented in randomized order under uniform lighting at a suitable temperature in an odor-free environment. Panelists rinsed their mouths with water and rested briefly between samples to minimize sensory carryover.

Each sample was evaluated in triplicate, color, aroma, taste, and texture were rated on a 10-point scale (Table 1; 0 = completely unacceptable, 10 = excellent sensory quality), with weights of 15%, 30%, 30%, and 25%, respectively. Overall acceptability was evaluated independently based on the panelists’ overall eating experience. The weighted sensory score (S) was calculated as follows: S_100_ = 10 × (0.15C + 0.30A + 0.30T + 0.25X), where C, A, T, and X represent the scores for color, aroma, taste, and texture, respectively.

Given the relatively high dietary fiber content of *P. cornutum*, texture evaluation emphasized tenderness, ease of chewing, perceived fibrousness, chewing resistance, and tissue integrity rather than crispness alone.

### 2.3. Conventional Physicochemical Indicators

Conventional physicochemical parameters (e.g., pH, titratable acidity, NaCl content) were determined in accordance with Chinese National Standards. The pH of the pickling brine was measured using a calibrated pH meter. TA, NaCl content, nitrite content, reducing-sugar content, amino-N content, vitamin C (Vc) content, and moisture were determined using homogenized tissue samples.

Moisture was determined gravimetrically after drying at 105 ± 5 °C. TA (expressed as lactic acid equivalents) and NaCl contents were determined by titration according to GB 12456-2021 [13] and GB 5009.44-2016 [14], respectibely, Vc content and amino-N content was determined also by titration according to GB 5009.86-2016 [15] and GB 5009.235-2016 [16], respectively. Nitrite content (as mg NaNO_2_/kg of fresh weight) and reducing-sugar content were determined by UV-visible spectrophotometry following GB 5009.33-2016 [17] and GB 5009.7-2016 [18], respectively. Each sample was measured in triplicate.

### 2.4. E-Nose Measurement

E-nose measurement was determined according to the method [19] with a few modifications. Overall aroma profiles of *P. cornutum* pickles at different pickling stages were evaluated using a portable electronic nose system (PEN3, Airsense Analytics GmbH, Schwerin, Germany), which consists of ten metal oxide semiconductor sensors: W1C (benzene), W5S (nitrogen oxides), W3C (ammonia-based compounds), W6S (Hydride), W5C (short-chain alkane aromatic components), W1S (methyl compounds), W1W (sulphur compounds), W2S (alcohols, aldehydes and ketones), W2W (Aromatic components, organic sulfides compounds), and W3S (long-chain alkanes components).

A 1.5 g sample was placed in a sealed 20 mL headspace vial and equilibrated at 60 °C for 20 min. A stainless-steel sampling needle fitted with a 0.22-micron membrane filter was then inserted into the vial headspace. Instrument settings were as follows: clearing time, 60 s; measurement time, 150 s; waiting time, 5 s; cavity air flow rate, 0.4 L/min; and injection flow rate, 0.016 L/min. Five parallel measurements were performed for each sample, and the stable sensor responses recorded at the end of the measurement period were used for subsequent analyses.

### 2.5. Analysis of Volatile Compounds by Hs-Gc-Ims

VOCs in *P. cornutum* pickle samples were analyzed using a FlavourSpec^®^ gas chromatography–ion mobility spectrometry system (G.A.S., Dortmund, Germany). Volatile compounds were determined according to the method [8]. A 1.5 g sample was placed in a 20 mL headspace vial and incubated at 60 °C for 20 min. Then, 500 μL of headspace gas was automatically injected using a heated syringe maintained at 85 °C. VOCs were separated on an MXT-5 capillary column (15 m × 0.53 mm × 1 µm) maintained at 60 °C. High-purity nitrogen (≥99.999%) served as the carrier gas and drift gas. The carrier gas flow program was maintained at 2 mL/min for 0–2 min, increased to 100 mL/min over 2–20 min, and maintained at 100 mL/min until 30 min.

An external n-ketone mixture (C4~C9, Sinopharm Chemical Reagent Beijing Co., Ltd., Beijing, China) was used to calculate retention indices. VOCs were assigned by matching the retention index (RI) and normalized drift time of standards in the GC-IMS library. Peak areas were used for semi-quantitative comparison of relative abundance. 3D spectra, 2D topographic plots, differential spectra, and fingerprint plots were generated using VOCal software (version 0.4.35, G.A.S., Dortmund, Germany). Each sample was measured in triplicate.

### 2.6. Statistical and Multivariate Analyses

Results are expressed as mean ± standard deviation (SD). Statistical differences among pickling times were tested by one-way analysis of variance followed by Tukey’s multiple comparison in SPSS 19.0 (IBM SPSS Inc., Chicago, IL, USA), with *p* < 0.05 considered statistically significant. PCA and PLS-DA were performed using SIMCA software (14.1, Sartorius Stedim Data Analytics, Umeå, Sweden), and graphs were generated using Origin. For adjacent-stage E-nose comparisons, log2 fold change was calculated as log2FC = log2(Rt2/Rt1), where Rt1 and Rt2 represent the mean stable sensor responses at two adjacent sampling times. Spearman correlation analysis was performed in R, and heatmaps and network visualizations were also generated in R.

## 3. Results

### 3.1. Sensory Evaluation Results

**Figure 2:**
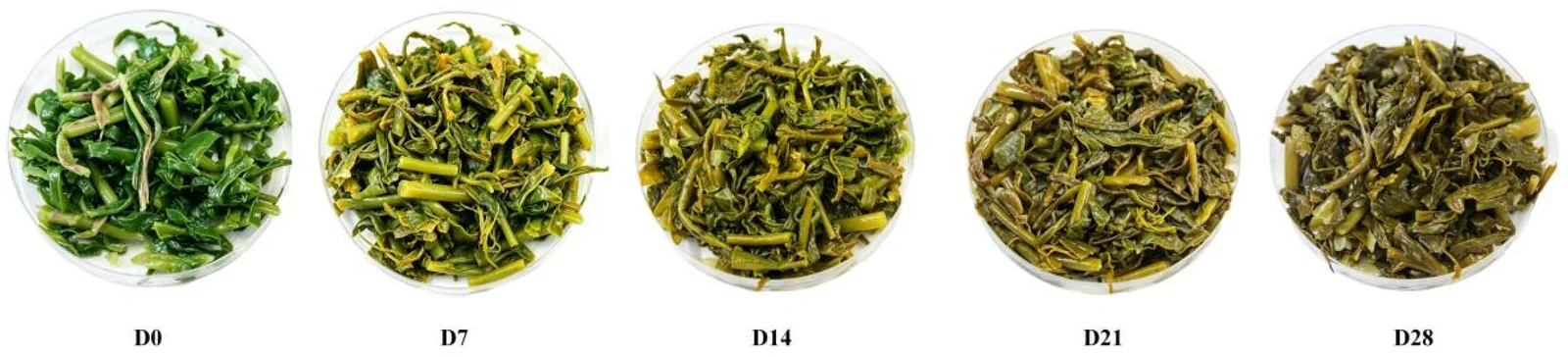
Changes in the visual appearance of *P. cornutum* during pickling.

Pickling markedly changed the sensory profile of *P. cornutum* (Table 2 and Figure 2). The weighted sensory score increased from 42.75 (D0) to 81.84 (D21) and 83.98 (D28). Aroma, taste, texture, and overall acceptability increased over time, whereas color scores decreased. No significant differences were observed between D21 and D28 for aroma, taste, texture, or overall acceptability (*p* > 0.05), indicating that panel-assessed sensory quality approached a plateau during the late stage.

**Figure 3:**
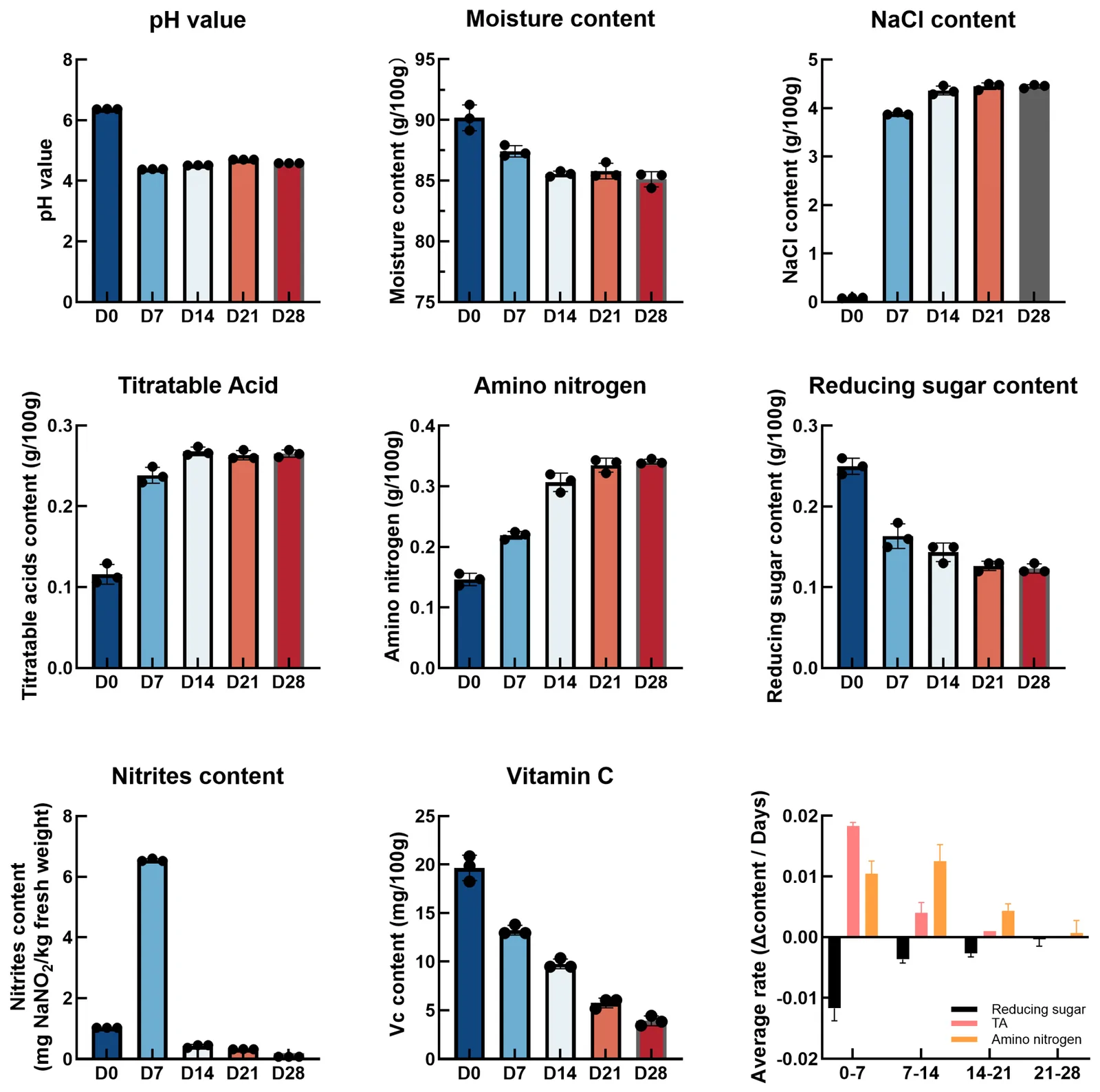
Changes in physicochemical parameters during 28 days of *P. cornutum* pickling (*n* = 3).

As shown in Figure 3, the appearance of *P. cornutum* changed progressively from bright green at D0 to yellowish-green/olive-green at D7–D14 and darker olive-green/yellowish-brown green at D21–D28. Consistent with the visual change, the color score declined from 9.25 at D0 to 6.23 at D28. This shift may be related to the acidic environment during pickling triggers chlorophyll degradation to form pheophytin derivatives. Meanwhile, carotenoids are relatively stable under these acidic conditions, and their color gradually becomes dominant, resulting in the transition of plant tissues from bright green to deep green or olive brown [20,21].

In contrast to color changes, aroma and taste continuously improved during pickling. The D0 sample mainly retains the original pungent and spicy flavor of *P. cornutum*, with initial scores of 3.27 and 3.75, respectively. As pickling proceeded, these harsh sensory attributes faded, replaced by a balanced combination of acidity, saltiness, umami and typical *P. cornutum* flavor profile. By days 21–28, a mature, complex and well-balanced pickled flavor was fully developed. Texture is another critical determinant of sensory acceptability. The D0 sample displayed high chewing resistance owing to its fibrous tissue structure. With increasing pickling time, tissue softened progressively, reducing both fiber content and chewing toughness. Samples collected at D21 and D28 presented tender, chewable textures while retaining favorable structural integrity, with a texture score reaching 8.62 and 8.74, respectively. These results indicate that moderate tissue softening effectively improved the edible quality of pickled *P. cornutum*, without excessive texture deterioration within the experimental period.

Overall acceptability increased gradually during pickling and stabilized during the course of 21–28 days. No significant difference in overall acceptability was found between the D28 and D21 samples, despite the D28 sample’s slightly deeper color (*p* > 0.05). This suggests that consumers’ perceptions of overall quality at maturity reflect a balanced integration of color, aroma, flavor, and texture rather than changes in any one attribute. Considering all sensory parameters together, pickled *P. cornutum* reaches its optimal state of sensory maturity between days 21–28, when aroma, flavor, and texture have reached harmonious balance and overall sensory quality is consistently high and steady.

### 3.2. Conventional Physicochemical Evolution

To characterize physicochemical changes during pickling, pH, moisture, NaCl content, reducing-sugar content, TA, amino-N content, Vc content, and nitrite content, were monitored throughout the 28-day process (Figure 3).

As pickling progressed, the pH of the brine decreased initially and then stabilized. Moisture gradually decreased, whereas NaCl content increased rapidly from 0.085 ± 0.004 g/100 g at D0 to 3.91 ± 0.05 g/100 g at D7 and then approached 4.37 ± 0.08 g/100 g. This early increase is consistent with rapid salt penetration and solute exchange between the *P. cornutum* tissue and brine. Such changes may affect water activity, enzyme function, and the partitioning of volatile compounds [22,23,24]. Consistent with observations reported for other fermented vegetables, Vc content decreased throughout pickling, consistent with the susceptibility of ascorbic acid to processing-related oxidation and degradation [25]. Nitrite content reached 6.53 mg NaNO_2_/kg fresh weight at D7, decreased to 0.44 mg NaNO_2_/kg at D14, and further declined to 0.075 mg NaNO_2_/kg at D28.

Reducing-sugar content decreased throughout pickling, whereas TA increased and amino-N content rose progressively, with the latter changing more slowly after day 14. Interval-specific change rates confirmed that the first 7 days had the largest bulk physicochemical shifts: reducing sugar changed at −0.012 g/100 g/day, the TA accumulation rate reached +0.014 g/100 g/day, and the amino-N content showed its fastest increase. Thereafter, the rates of change decreased, suggesting lower substrate availability and a gradual approach to physicochemical equilibrium.

Overall, physicochemical indicators significantly differed among different pickling stages. Dynamic changes in pH, TA, and other quality indicators reflected the progression of fermentation and substrate degradation during *P. cornutum* pickling. The variation trends of physicochemical properties corresponded well with the changes in sensory characteristics, demonstrating that physicochemical transformation was the internal basis for sensory quality formation.

### 3.3. Stage-Dependent E-Nose Responses

**Figure 4:**
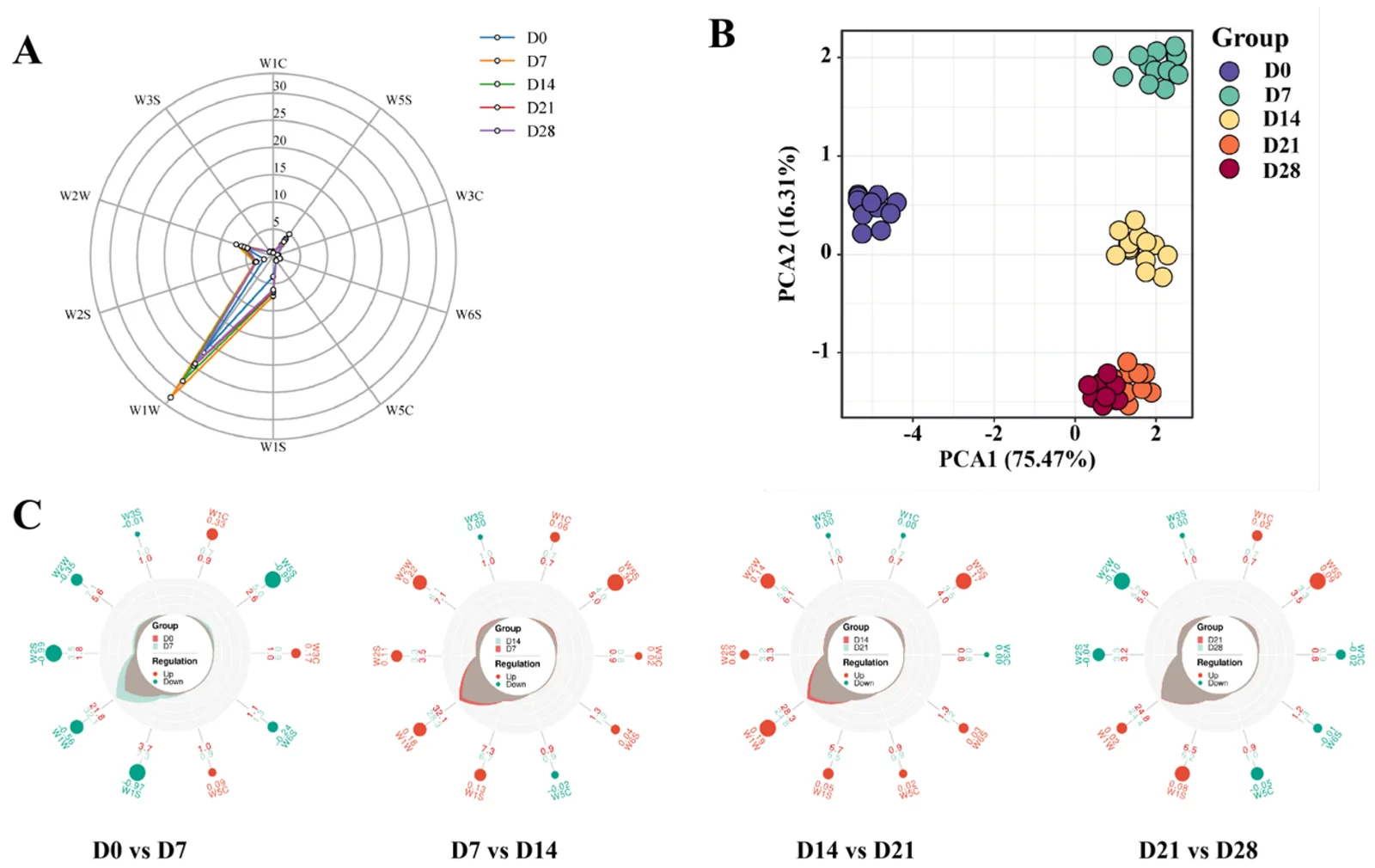
E-nose analysis of *P. cornutum* pickles at different pickling times. (**A**) Radar map; (**B**) PCA plot; (**C**) Adjacent-stage log2 fold changes in sensor responses.

E-nose technology has been widely applied for rapid characterization of aroma profiles in pickled foods because of its high sensitivity to volatile compounds and its ability to distinguish aroma differences among samples [26,27]. The radar plot showed stage-dependent changes in the sensor-response pattern of *P. cornutum* (Figure 4A). W2W and W1W displayed relatively high responses compared with most other sensors throughout pickling, indicating prominent responses in sensor channels commonly associated with sulfur- and aromatic-related volatiles.

PCA further differentiated the E-nose profiles (Figure 4B). The first two principal components explained 91.78% of the total variance (PC1 = 75.47%, PC2 = 16.31%), indicating that the PCA model adequately represented the aroma information captured by the E-nose system. Samples from D0, D7, and D14 formed separated clusters, whereas D21 and D28 showed overlap, indicating that the global patterns became more similar during the late stage. This late-stage similarity paralleled the absence of significant differences in overall sensory acceptability between D21 and D28.

To further examine temporal changes, adjacent-stage sensor responses were compared using log_2_FC analysis (Figure 4C). During the early pickling stage (first 7 days), sensor responses of the *P. cornutum* samples changed most dramatically, particularly evident in W1S, W1W, W2S, W5S, and W2W, indicating rapid remodeling of the volatile-response pattern. These changes may be associated with intensive biochemical and microbial activities driving the release and transformation of aroma-related compounds. Changes became smaller from days 7 to 21, and only minor fluctuations were observed from days 21 to 28 (|log_2_FC| < 0.1), indicating relative stabilization of the overall E-nose response. Overall, the sensor array clearly captured the transition from a dynamic profile characterized by intense early evolution to a mature and stable profile in the later stages of *P. cornutum* fermentation.

The overall odor response characteristics of *P. cornutum* samples at different pickling stages can be well distinguished by E-nose. Specifically, substantial flavor evolution occurs during the early and middle stages of pickling, whereas the odor profile stabilizes in the later stages. This is consistent with the results of sensory evaluation and physicalchemical indicators.

### 3.4. Dynamic VOCs Remodeling Revealed by HS-GC-IMS

**Figure 5:**
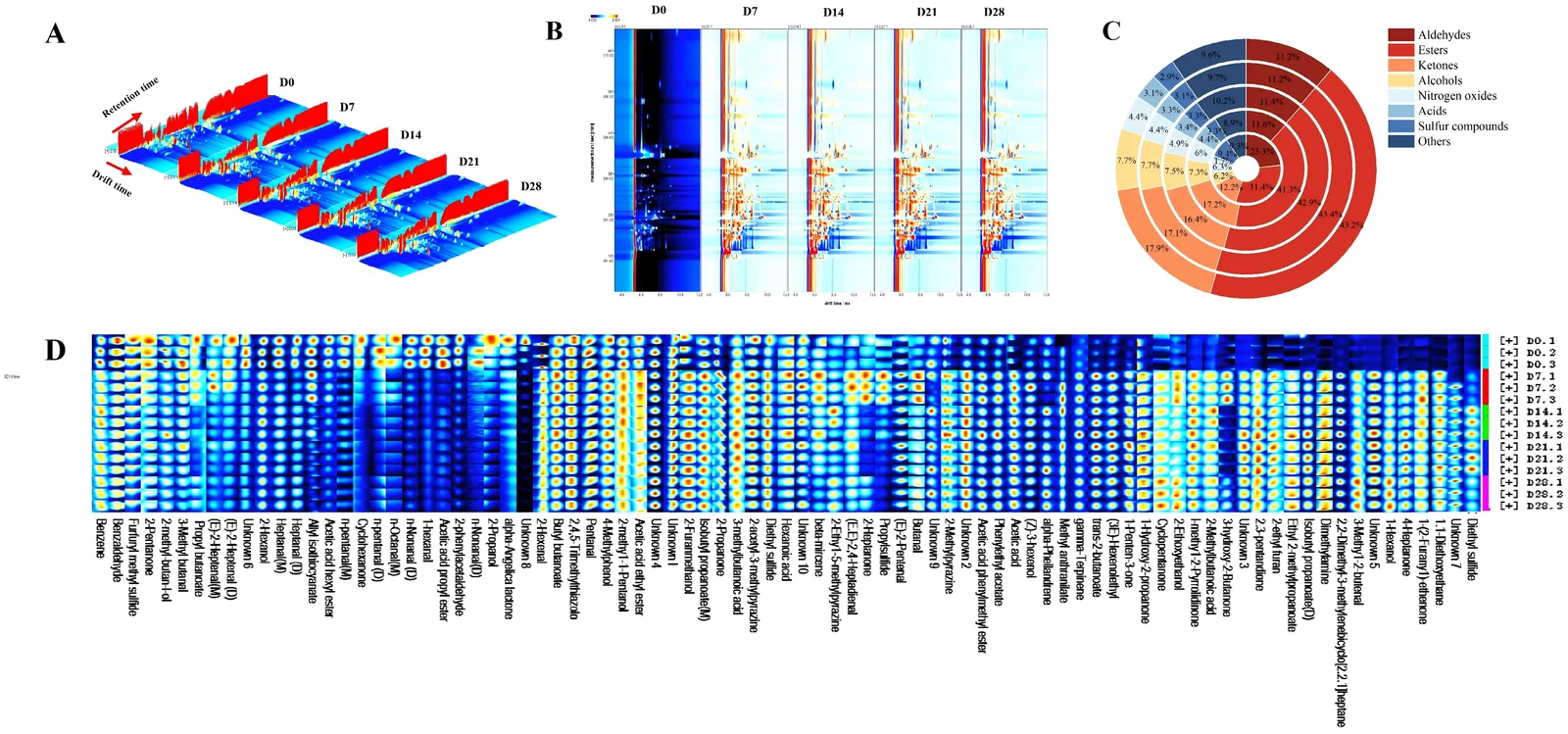
HS-GC-IMS spectra and fingerprints in *P. cornutum* at different pickling times. (**A**) 3D topographic plot; (**B**) differential top-view plots using D0 as the reference; (**C**) Relative contents of VOC classes; (**D**) Gallery fingerprint.

HS-GC-IMS was used to investigate the dynamic evolution of VOCs during *P. cornutum* pickling. The three-dimensional topographic plots display migration time, retention time, and signal intensity along the X-, Y-, and Z-axes, respectively. Although the overall signal distribution was broadly similar among samples, clear stage-dependent differences in signal intensity were observed (Figure 5A). Differential spectra using D0 as the reference showed that several signals decreased or disappeared, while others increased during pickling (Figure 5B). Most signals occurred at retention times of approximately 100–400 s and drift times of 1.0–2.0 s.

A total of 84 characteristic signal peaks were detected. Based on retention-index and normalized-drift-time matching against the GC-IMS library, 75 VOCs were successfully identified: 19 aldehydes, 14 esters, 11 ketones, 9 alcohols, 4 organic acids, 5 sulfur-containing compounds, 4 nitrogen-containing compounds, and 9 other compounds (Table S1). It is worth noting that 24 compounds were detected in both monomeric and dimeric forms. In GC-IMS, the same compound can generate monomer and higher-order cluster signals when analyte concentration and ion-molecule association favor proton-bound complexes; comparable monomer/dimer patterns have been reported in recent GC-IMS food-aroma studies [28,29].

Perform normalization comparison based on peak areas (Figure 5C), aldehydes, sulfur-containing compounds, and nitrogen-containing signals generally decreased with pickling, whereas esters, ketones, and organic acids tended to increase. The relative content of aldehydes decreased from 23.33% at D0 to 11.59% at D7 and then changed comparatively little, while the ketone signal proportion increased from 12.21% to 17.23% over the same interval before approaching a plateau. The timing of these class-level changes was consistent with the D0–D7 physicochemical transition and the largest E-nose response shift.

To characterize individual VOC-signal trajectories, the gallery fingerprint was constructed (Figure 5D). Based on the profiles, VOCs were broadly categorized into three groups: the first exhibited a continuous decreased during *P. cornutum* pickling, exemplified by allyl isothiocyanate (a pungency-associated *Brassicaceae* volatile) and 2-hexanol; the decline in allyl isothiocyanate was temporally consistent with the increase in aroma acceptability and the attenuation of pungent sensory characteristics. The second group consisted of compounds showing relatively stable abundances, such as butyl butanoate and 4-methylphenol. The third group accumulated during pickling, including ethyl acetate, isobutyl propionate, 2-furanmethanol, 1-penten-3-one, methyl anthranilate, diethyl sulfide, 1-hexanol, and 2,3-pentanedione. These compounds contribute pleasant aromas, including fruity, sweet, and floral notes, and their gradual accumulation may play a key role in the formation of the characteristic flavor of pickled *P. cornutum* products.

**Figure 6:**
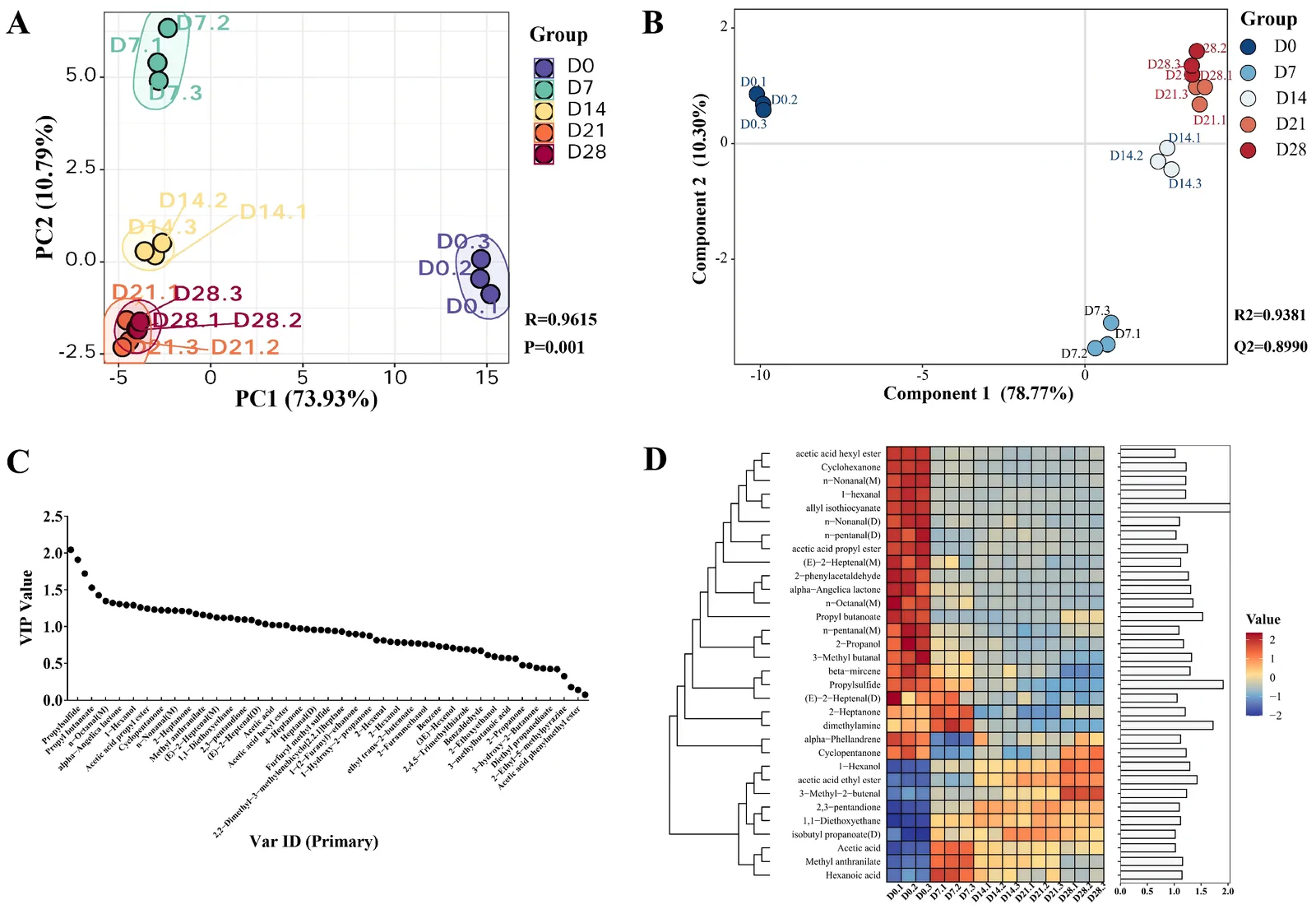
Multivariate analysis of HS-GC-IMS results in *P. cornutum* at different pickling times. (**A**) PCA score plot; (**B**) PLS-DA score plot; (**C**) VIP values; and (**D**) Heatmap of differential VOCs with VIP > 1.

To clarify the flavor differences and classification effects of *P. cornutum* samples at different pickling stages, a multivariate statistical analysis method was employed. First, unsupervised PCA was performed on *P. cornutum* samples at five pickling time points. PCA results (PC1 and PC2 explaining 73.93% and 10.79% of variance, respectively, Figure 6A) showed obvious separation among samples from different pickling periods, while overlap was observed between the D21 and D28 groups. As PCA does not incorporate category labels during modeling, its discriminative capacity is limited. Supervised PLS-DA was therefore applied to amplify inter-group differences and establish a discrimination model. PLS-DA also separated the stages (Figure 6B), and the 200-permutation test yielded R2 and Q2 intercepts of 0.215 and −1.0108, respectively. Based on the criterion of VIP > 1.0, 33 key differential VOCs were successfully screened (Figure 6C), including allyl isothiocyanate, 2-heptanone, propyl sulfide, and propyl butanoate, which were regarded as the core characteristic substances distinguishing different pickling stages of *P. cornutum*. The heatmap of the 33 differential VOCs showed two broad temporal clusters (Figure 6D).

The heatmap of the 33 differential VOCs showed two broad temporal clusters. The signals assigned to allyl isothiocyanate, propyl sulfide, alpha-phellandrene, hexyl acetate and n-hexadecanal were relatively high at D0 and decreased during pickling, consistent with attenuation of the early pungent/green sensory profile. In contrast, the signals including acetic acid and 3-methyl-2-butenaldehyde increased toward the later stages. These trajectories may help distinguish the pre-fermentation pretreated sample from later-stage samples. These changes may reflect multiple pathways involving lipid oxidation, amino acid metabolism, carbonyl reduction, glucosinolate-related reactions, and possible microbial metabolism [3,30,31]. Aldehydes can arise from the oxidation of unsaturated fatty acids; ketones may originate through several routes, including lipid oxidation and amino acid catabolism; alcohols can be generated through fatty acid-derived reactions and carbonyl reduction; and sulfur-containing compounds may arise from glucosinolate hydrolysis or pathways related to sulfur amino acids [32].

Taken together, HS-GC-IMS provided compound-resolved analytical support for the stage-dependent aroma trajectory detected by E-nose. The decrease in several aldehydes and sulfur-related signals, together with increases in esters, ketones, and organic acid related signals, was consistent with the panel-observed shift from a pungent early profile toward a more acceptable late-stage pickled aroma, and this agreement is associative and temporal.

### 3.5. Correlation Analysis

**Figure 7:**
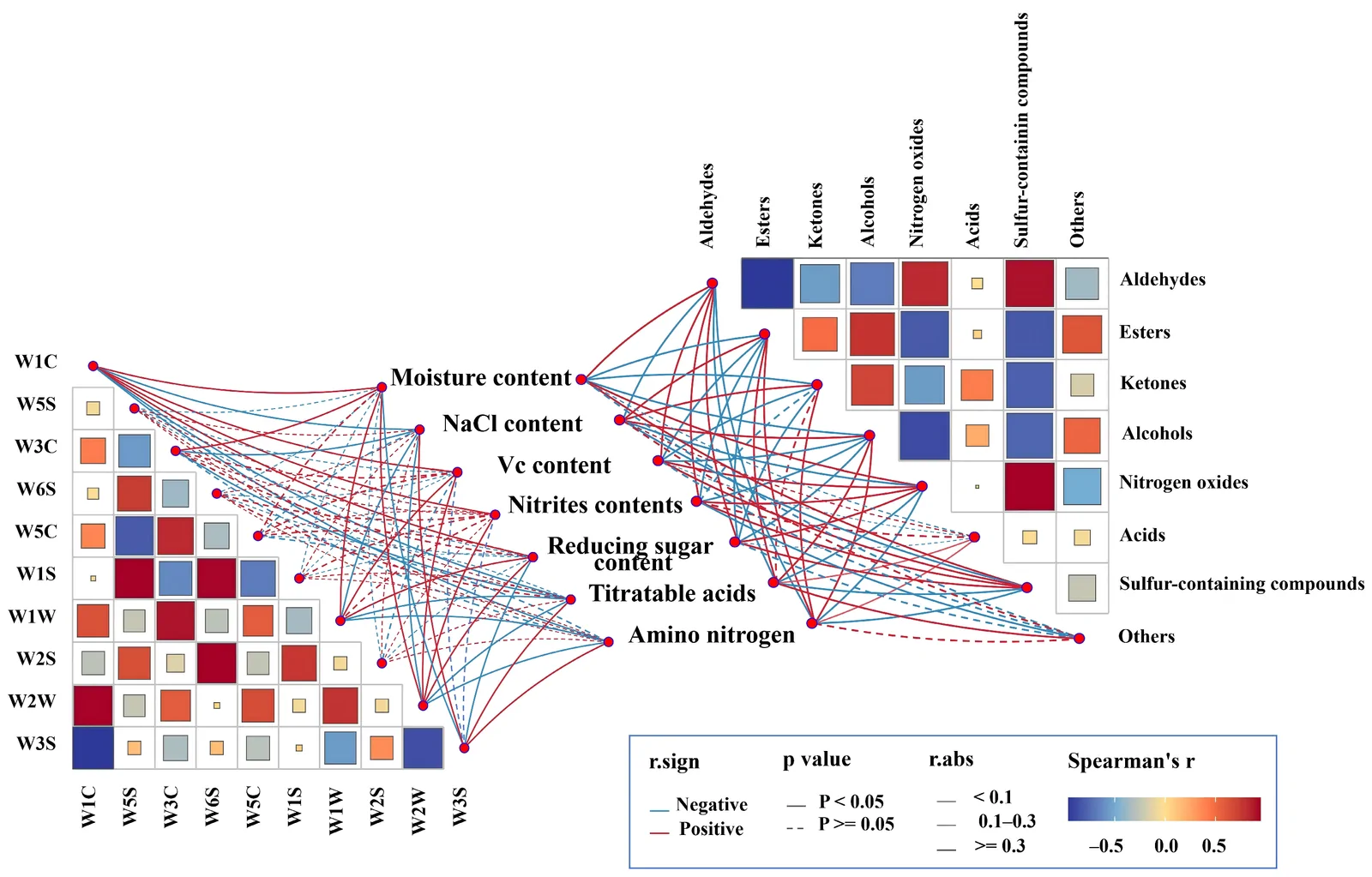
Spearman correlation network and matrix linking physicochemical parameters, E-nose sensor responses, and VOC classes.

To explore associations between physicochemical changes and aroma-related measurements during *P. cornutum* pickling, Spearman correlation were calculated among physicochemical variables, E-nose responses, and VOC classes. The correlation network (Figure 7) illustrated the associations between conventional physicochemical indicators, VOC classes, and E-nose signals, with blue and red lines indicating negative and positive correlations, respectively, and line thickness indicating correlation strength.

For volatile components, TA and amino-N were positively associated with the relative contents of esters and alcohols (*p* < 0.01), whereas reducing sugar was negatively associated with these compounds (*p* < 0.05), and NaCl was also negatively associated with sulfur-containing volatiles. These results indicate that acid accumulation, amino-nitrogen increase, substrate consumption, and volatile remodeling co-varied during *P. cornutum* pickling. For E-nose responses, TA and amino-N were positively correlated with W3S and negatively correlated with W1C, W1W, and W2W. Moisture showed positive associations with W1C, W3C, W1W, and W2W and a negative associations with W3S, whereas NaCl generally showed the opposite pattern. The observed decrease in reducing sugar is consistent with substrate utilization during natural pickling, while increasing NaCl and decreasing moisture coincided with lower sulfur-containing volatile signals. These temporal and statistical associations provide a link between matrix evolution and aroma-related measurements of *P. cornutum* pickling, and may provide a theoretical basis for flavor regulation in pickled *P. cornutum*.

**Figure 8:**
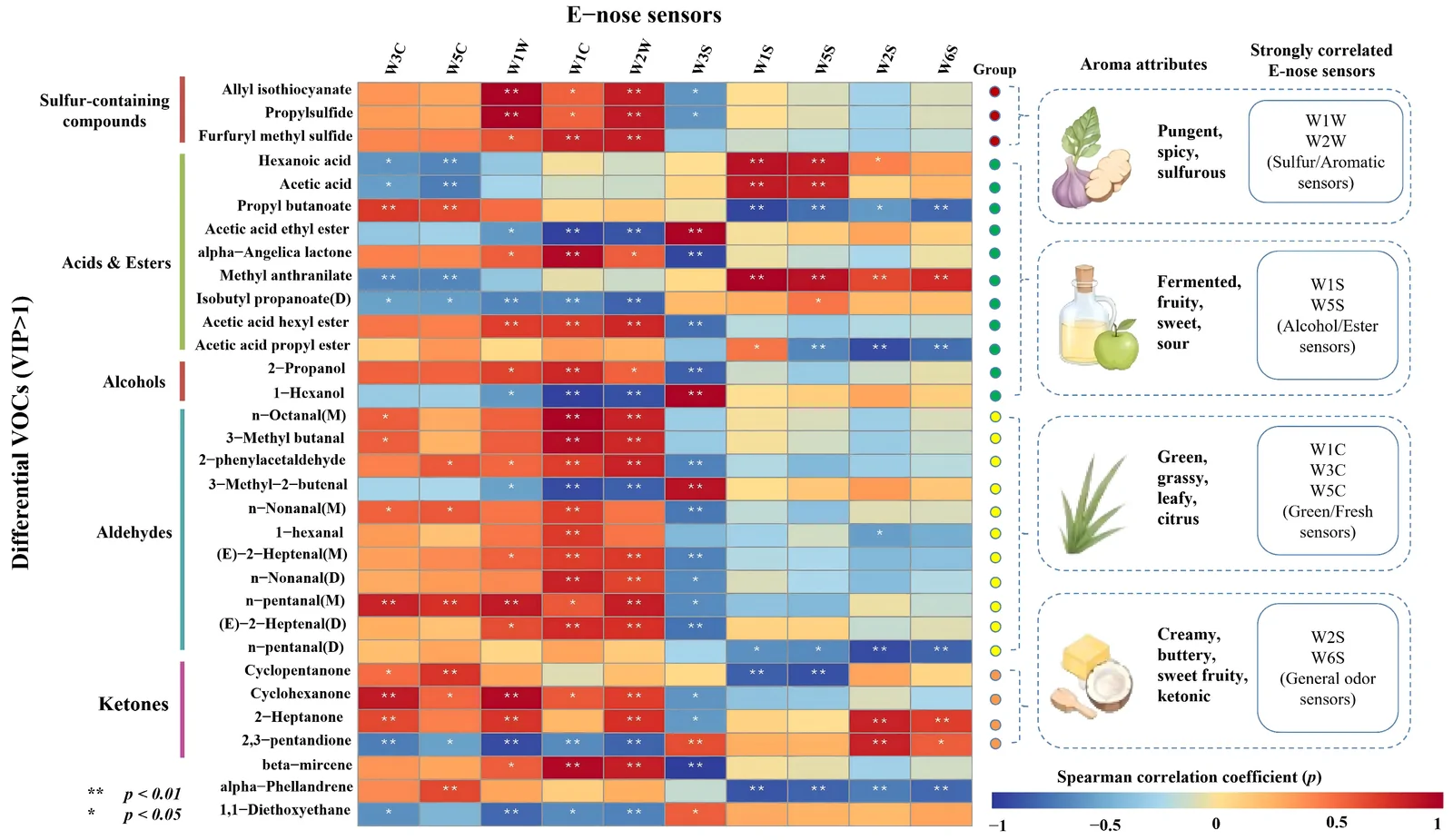
Spearman correlations between E-nose sensor responses and differential VOCs (VIP > 1).

Associations were examined between the sensor responses and the differential VOCs (Figure 8). Allyl isothiocyanate and propyl disulfide exhibited significant positive correlations with the W1W and W2W sensors (*p* < 0.01), consistent with the responsiveness of these sensor channels to sulfur-related volatiles, although the sensors remain non-specific. Several aldehydes (e.g., 1-hexanal and n-pentanal (M)) were associated with W3C and W5C, whereas acetic acid and ethyl acetate showed positive correlations with W1S and W5S. These correlations reinforce the analytical linkage between E-nose patterns and compound-level GC-IMS signals. As fermentation proceeds, the abundance of allyl isothiocyanate, a characteristic compound contributing pungent sensory notes, gradually decreases. This shift transforms the dominant aroma profile from the sharp, irritating odor observed in the D0 sample toward mild pickled flavors dominated by acids and alcohols at later stages, supported by the continuous accumulation of acids and esters during prolonged pickling.

Correlation analysis revealed significant correlations between the screened key volatile compounds, the E-nose response, and conventional physicochemical indicators. Many characteristic flavor substances were closely associated with aroma and overall acceptability, indicating that the dynamic accumulation of volatile components was the critical factor driving the quality variation in pickled *P. cornutum*.

## 4. Discussion

### 4.1. Quality Evolution and Pungency Attenuation During Pickling

Fermented-pickled vegetables gain their final sensory quality from the interplay among physicochemical transformation, substrate consumption, and dynamic accumulation of volatile flavor compounds during natural pickling. In this study, time-dependent changes in physicochemical indices, volatile profiles, and sensory attributes were characterized throughout the natural pickling of *P. cornutum*. Progressive acidification reshaped the tissue matrix and volatile metabolome, leading to continuous improvement and subsequent stabilization of sensory acceptability, consistent with reports on other *Brassicaceae* fermented vegetables [33,34,35,36].

Based on combined physicochemical, HS-GC-IMS, E-nose and sensory evidence, the whole pickling process was divided into three phases: an early rapid-transition phase (0–7 d), an intermediate maturation phase (7–21 d), and a late stable phase (21–28 d). Within the first 7 days, pH, TA, reducing sugars, amino-N, and nitrite shifted sharply, accompanied by pronounced E-nose drift and large-scale VOCs remodeling, and weighted sensory acceptability increased markedly, though full sensory maturation was not yet reached. A transient nitrite peak at day 7 was related to early nitrate-reduction activity, and its decline was attributed to progressive acidification [37,38]. Notably, the color scores declined while overall acceptability improved, indicating that a mature quality relied on comprehensive sensory performance rather than retention of fresh-green appearance alone, which could be partly explained by chlorophyll degradation under acidic pickling conditions [39,40].

Degradation of gucosinolates is a core event governing pungency evolution in pickled *P. cornutum*. Microbial-driven acidification facilitates both myrosinase catalyzed and non-enzymatic hydrolysis of endogenous glucosinolates [41,42]. Isothiocyanates, the major pungency originating decomposition products of glucosinolates, are chemically unstable under low-pH fermentation environments and undergo further transformation, volatilization and degradation into less pungent sulfur-containing derivatives during prolonged pickling [43,44]. In the present study, the decline in allyl isothiocyanate occurred in parallel with increasing aroma scores and attenuation of pungent sensory characteristics. This consistency indicates that sulfur-containing volatiles constitute the key material basis for the pungent flavor of *P. cornutum* at the early pickling stage.

### 4.2. Volatile Flavor Remodeling and Flavor-Marker Identification

Acidification altered the metabolic pathways of the vegetable substrate and reshaped the volatile fingerprints [45]. Reducing sugars were rapidly consumed within the first 7 days as carbon sources for local microorganisms. Sugars, free amino acids and lipid-derived precursors were further catabolized into organic acids, ketones and aroma-active esters [46]. Accumulated organic acids not only built the typical sour taste, but also served as key precursors for ester-synthesis reactions. Aldehydes originating from lipid oxidation and Strecker degradation decreased during pickling, which could be explained by acid-promoted reduction–condensation reactions generating alcohols for subsequent esterification [47]. Progressive accumulation of esters and ketones enriched pickled and fruity notes, and correlation analysis revealed coordinated changes between physicochemical properties and volatile components during the pickling process of *P. cornutum*, but the underlying formation mechanism still needs to be further elucidated.

E-nose and HS-GC-IMS provided complementary odor information. The E-nose captured the global odor differences among pickling stages, and 75 VOCs belonging to aldehydes, alcohols, ketones, and esters were identified by GC-IMS, constituting the fundamental flavor framework of pickled *P. cornutum*. Plant-derived grassy and green-odor volatiles dominated early pickling samples and faded gradually, whereas fermentation-related flavor metabolites accumulated continuously and shaped the characteristic aromatic traits of mature products. PCA clearly separated the pickled samples according to their pickling stages, and PLS-DA screened 33 VOCs (VIP > 1) as core differential markers, which contributed to inter-sample flavor differences and could act as candidate biomarkers reflecting the flavor-maturation degree of pickled *P. cornutum*. These markers were positively correlated with aroma and overall acceptability but negatively with several native plant volatiles, demonstrating that dynamic VOC transformation was the intrinsic cause of sensory quality changes in pickled *P. cornutum*.

### 4.3. Practical Implications, Limitations and Future Perspectives

At 21–28 d, the main physicochemical indicators, sensory scores and overall E-nose signals reached a plateau. Nevertheless, subtle VOC differences between D21 and D28 implied that minor remodeling still proceeded, warning against judging pickling completion by a single analytical platform. Day 21 is recommended as a feasible *P. cornutum* pickling endpoint: further prolongation hardly improves comprehensive quality while raising risks of over-acidification, tissue softening and off-flavor formation. A stage-specific monitoring strategy is therefore proposed: early-stage surveillance should focus on safety-related physicochemical indices (pH/TA, salt, reducing sugars, nitrite), whereas the maturation phase benefits from combined sensory evaluation, E-nose screening and targeted VOC detection. Under the present conditions, 21–28 d represents the flavor-maturation window for naturally pickled *P. cornutum*.

This work systematically characterized the sensory-physicochemical-volatile evolution and screened representative flavor biomarkers, providing a theoretical basis for quality control and industrial production of pickled *P. cornutum*. However, microbial community dynamics were not analyzed herein, so direct causal links between specific taxa and flavor-metabolite transformation, as well as the precise pathways generating key flavor compounds, cannot be established. Future work could screen and validate phase-specific volatile markers and systematically clarify their relationships with sensory attributes. Meanwhile, future research integrating high-throughput microbial sequencing with targeted metabolomics will help unravel the microbial-driven flavor-forming mechanism of pickled *P. cornutum*.

## 5. Conclusions

This study integrated sensory evaluation, physicochemical indices, E-nose, HS-GC-IMS, and multivariate analyses to characterize the 28 days of natural *P. cornutum* pickling. The first 7 days were the principal physicochemical and overall aroma transition interval, with rapid salt uptake, pH decrease/TA increase, reducing-sugar depletion, amino-N increase, a transient nitrite peak, the largest E-nose changes, and marked changes in aldehydes-, ketones-, and sulfur-related signals. Sensory quality continued to improve beyond this early transition: the weighted sensory score increased from 42.75 at D0 to 81.84–83.98 at D21 and D28, while overall acceptability showed no significant difference between samples of D21 and D28. E-nose profiles also converged at D21 and D28, whereas HS-GC-IMS indicated continued variation in selected VOC-related signals. HS-GC-IMS detected 84 signal peaks and tentatively assigned 75 VOCs, and 33 differential VOCs contributed to stage discrimination. Correlation analysis identified associations among physicochemical variables, E-nose responses, and differential VOCs. Collectively, the results describe an early matrix transition, an intermediate period of continued sensory and volatile development, and a late-stage sensory/global-aroma plateau. During the natural pickling process of *P. cornutum*, a potential window of maturity can be defined as 21 to 28 days. In summary, these findings can provide a solid theoretical basis for the processing optimization and quality regulation of pickled *P. cornutum* products.

## Figures and Tables

**Table 1:** Sensory evaluation criteria for pickled *P. cornutum*.

Attribute	Weight (%)	Evaluation Criteria
Color	15	Naturalness and uniformity of color, gloss, and degree of abnormal browning. Higher scores indicate a natural and uniform appearance with minimal dullness, browning, or uneven coloration.
Aroma	30	Characteristic *P. cornutum* aroma, intensity and harmony of the pickled aroma, and absence of green, pungent, or other undesirable odors. Higher scores indicate strong, pleasant, well-balanced pickled *P. cornutum* aroma with minimal off-odors.
Taste	30	Intensity and balance of sourness, saltiness and umami typical of pickled *P. cornutum*; absence of obvious bitter, acrid, stale or unpleasant off-tastes. Higher scores represent well-balanced sour-salty taste, prominent umami, and no undesirable flavor defects.
Texture	25	Tenderness, ease of chewing, fibrous sensation, chewing resistance, and tissue integrity. Higher scores indicate a tender and easily chewable texture with appropriate structural integrity and without excessive hardness or mushiness.
Overall acceptability	—	Independently evaluated according to the overall eating experience, integrating color, aroma, taste, and texture.

**Table 2:** Sensory scores of *P. cornutum* during pickling (mean ± SD, *n* = 8).

Samples	Color	Aroma	Taste	Texture	Overall Acceptability	Weighted Sensory Score/100
D0	9.25 ± 0.43 ^a^	3.27 ± 0.74 ^a^	3.75 ± 0.46 ^a^	3.00 ± 0.76 ^a^	5.38 ± 0.92 ^a^	42.75 ± 3.78 ^a^
D7	8.00 ± 0.54 ^b^	6.12 ± 0.60 ^b^	6.38 ± 0.51 ^b^	4.33 ± 0.50 ^b^	6.50 ± 0.53 ^b^	60.44 ± 4.06 ^b^
D14	7.25 ± 0.89 ^b^	7.61 ± 0.49 ^c^	7.12 ± 0.34 ^b^	7.45 ± 0.63 ^c^	7.23 ± 0.41 ^b^	72.94 ± 3.63 ^c^
D21	6.39 ± 0.52 ^c^	8.38 ± 0.74 ^c^	8.49 ± 0.53 ^c^	8.62 ± 0.54 ^d^	8.38 ± 0.52 ^c^	81.84 ± 3.96 ^d^
D28	6.23 ± 0.46 ^c^	8.62 ± 0.52 ^d^	8.88 ± 0.64 ^c^	8.74 ± 0.71 ^d^	8.50 ± 0.50 ^c^	83.98 ± 1.21 ^d^

Ps: Sensory attributes were scored on a 10-point scale. A higher score represents better sensory performance. Different superscript letters (^a–d^) within the same row indicate significant differences among pickling times (one-way ANOVA followed by Tukey’s test, *p* < 0.05); identical letters denote non-significant differences.

## Data Availability

The original contributions presented in this study are included in the article/Supplementary Materials. Further inquiries can be directed to the corresponding author.

## Supplementary Materials

The following supporting information can be downloaded at: https://www.mdpi.com/article/10.3390/foods15193513/s1, Table S1: Qualitative and relative quantitative results of volatile components in pickled Pugionium samples with different times based on HS/GC-IMS.
