# Supplementary material for: Quality and Flavor Evolution of Pugionium cornutum (L.) Gaertn. During Natural Pickling: Insights from Sensory and Physicochemical Characterizations
Source: Foods. 2026 Oct 1;15(19):3513. doi: 10.3390/foods15193513 (PMC13665313; doi:10.3390/foods15193513)
Supplement: Supplementary file 1 [file foods-15-03513-s001.zip › foods-4607431-supplementary.pdf]

Table S1 Qualitative and relative quantitative results of volatile components in pickled *Pugionium* samples with different times based on HS/GC-IMS

No.	Compound	CAS#	Formula	MW	RI	Rt [sec]	Dt [a.u.]	Average of relative content（%）				
								D0	D7	D14	D21	D28
**Aldehydes (19)**												
14	n-Nonanal(M)	C124196	$C_9H_{18}O$	142.2	1107.4	518.511	1.4887	2.34±0.03	0.69±0.02	0.59±0.04	0.51±0.01	0.58±0.06
12	n-Nonanal(D)	C124196	$C_9H_{18}O$	142.2	1106.5	516.959	1.9249	0.20±0.00	0.07±0.00	0.07±0.01	0.06±0.01	0.06±0.00
29	2-Phenylacetaldehyde	C122781	C_8H_8O	120.2	1042.9	417.169	1.2643	1.04±0.16	0.23±0.02	0.23±0.01	0.20±0.01	0.21±0.02
20	(E)-2-Heptenal(D)	C18829555	$C_7H_{12}O$	112.2	956.6	319.155	1.6590	0.33±0.10	0.30±0.07	0.20±0.00	0.20±0.03	0.18±0.02
17	n-Octanal(M)	C124130	$C_8H_{16}O$	128.2	1006.4	368.874	1.8069	0.22±0.04	0.08±0.01	0.06±0.01	0.06±0.00	0.05±0.00
50	Heptanal(D)	C111717	$C_7H_{14}O$	114.2	898.4	271.185	1.6966	0.33±0.04	0.14±0.01	0.19±0.01	0.18±0.01	0.20±0.01
35	Heptanal(M)	C111717	$C_7H_{14}O$	114.2	898.5	271.283	1.3495	1.07±0.08	0.44±0.02	0.55±0.03	0.50±0.03	0.53±0.02
32	(E,E)-2,4-Heptadienal	C4313035	$C_7H_{10}O$	110.2	1013.5	377.790	1.1965	0.21±0.05	0.20±0.03	0.16±0.01	0.17±0.02	0.17±0.01
39	2-Hexenal	C505577	$C_6H_{10}O$	98.1	844.3	238.579	1.1816	0.69±0.07	0.38±0.04	0.37±0.06	0.34±0.01	0.35±0.00
72	1-Hexanal	C66251	$C_6H_{12}O$	100.2	788.2	209.569	1.5615	1.35±0.06	0.34±0.03	0.31±0.04	0.33±0.01	0.29±0.02
9	n-Pentanal(D)	C110623	$C_5H_{10}O$	86.1	687.9	169.493	1.4238	1.72±0.23	0.50±0.00	0.59±0.06	0.61±0.05	0.54±0.02
69	n-Pentanal(M)	C110623	$C_5H_{10}O$	86.1	694.2	171.692	1.1913	0.17±0.01	0.07±0.01	0.05±0.00	0.04±0.01	0.06±0.00
21	Butanal	C123728	C_4H_8O	72.1	579	142.602	1.2874	0.54±0.02	0.72±0.08	0.61±0.02	0.63±0.04	0.72±0.00
25	(E)-2-Pentenal	C1576870	C_5H_8O	84.1	743.4	190.509	1.3576	1.21±0.09	0.95±0.12	0.87±0.10	1.01±0.02	0.86±0.02
26	Pentanal	C110623	$C_5H_{10}O$	86.1	745.9	191.499	1.4130	3.24±0.02	1.74±0.10	1.80±0.06	1.81±0.11	1.80±0.02
59	3-Methyl-2-butenal	C107868	C_5H_8O	84.1	782.6	206.966	1.3427	0.35±0.01	0.47±0.03	0.48±0.04	0.52±0.01	0.69±0.01
36	(E)-2-Heptenal(M)	C18829555	$C_7H_{12}O$	112.2	955.7	318.343	1.2586	0.52±0.08	0.22±0.06	0.18±0.01	0.17±0.02	0.16±0.01
3	3-Methyl butanal	C590863	$C_5H_{10}O$	86.1	636.1	156.126	1.1974	3.30±0.44	1.71±0.24	1.39±0.07	1.41±0.12	1.10±0.06
28	Benzaldehyde	C100527	C_7H_6O	106.1	931.1	297.164	1.1553	3.81±0.30	2.37±0.11	2.55±0.12	2.39±0.08	2.50±0.03
**Esters (14)**												
2	Phenylethyl acetate	C103457	$C_{10}H_{12}O_2$	164.2	1209.3	730.950	1.3293	3.62±0.62	3.60±0.14	3.55±0.71	3.07±0.08	3.49±0.09
13	Acetic acid phenylmethyl ester	C140114	$C_9H_{10}O_2$	150.2	1168.7	637.459	1.3174	3.42±0.34	4.00±0.03	3.85±0.67	3.31±0.05	3.93±0.05
30	Diethyl propanedioate	C105533	$C_7H_{12}O_4$	160.2	1059.8	441.553	1.2488	1.72±0.37	1.39±0.05	1.43±0.14	1.29±0.03	1.41±0.03
5	Methyl anthranilate	C134203	$C_8H_9NO_2$	151.2	1322.8	1071.63	1.2597	12.43±3.38	15.71±4.61	14.42±3.49	15.18±1.32	13.28±0.39
31	Acetic acid hexyl ester	C142927	$C_8H_{16}O_2$	144.2	1005.8	368.090	1.4211	1.72±0.11	0.71±0.01	0.64±0.03	0.61±0.03	0.62±0.04
63	Propyl butanoate	C105668	$C_7H_{14}O_2$	130.2	886.5	263.029	1.2707	0.31±0.04	0.07±0.00	0.06±0.01	0.07±0.00	0.13±0.00

No.	Compound	CAS#	Formula	MW	RI	Rt [sec]	Dt [a.u.]	Average of relative content（%）				
								D0	D7	D14	D21	D28
52	Isobutyl propanoate(D)	C540421	C_7H_{14}O2	130.2	855.5	244.867	1.7124	0.11±0.04	0.28±0.04	0.29±0.06	0.34±0.01	0.29±0.02
64	Isobutyl propanoate(M)	C540421	$C_7H_{14}O_2$	130.2	854.8	244.488	1.2848	0.89±0.08	0.65±0.04	0.65±0.01	0.66±0.03	0.66±0.02
27	Ethyl trans-2-butenoate	C623701	$C_6H_{10}O_2$	114.1	833.6	232.746	1.5717	0.31±0.02	0.54±0.05	0.63±0.04	0.61±0.02	0.56±0.02
74	Acetic acid ethyl ester	C141786	$C_4H_8O_2$	88.1	591.9	145.557	1.3370	2.81±0.41	4.99±0.48	7.26±0.46	8.29±0.14	8.92±0.24
68	Ethyl 2-Methylpropanoate	C97621	$C_6H_{12}O_2$	116.2	744.4	190.900	1.5698	0.16±0.03	0.35±0.03	0.37±0.04	0.38±0.01	0.40±0.00
8	Acetic acid propyl ester	C109604	$C_5H_{10}O_2$	102.1	705.6	175.894	1.1621	2.28±0.15	0.37±0.02	0.54±0.04	0.52±0.02	0.45±0.00
4	Butyl butanoate	C109217	$C_8H_{16}O_2$	144.2	987.3	347.755	1.3393	2.36±0.11	1.70±0.12	1.56±0.03	1.46±0.04	1.38±0.03
61	Alpha-Angelica lactone	C591128	$C_5H_6O_2$	98.1	880.4	259.391	1.1242	0.49±0.07	0.20±0.02	0.14±0.01	0.13±0.00	0.12±0.01
	**Ketones (11)**											
48	1-(2-Furanyl)-ethanone	C1192627	$C_6H_6O_2$	110.1	898.9	271.582	1.4414	0.08±0.01	0.18±0.01	0.18±0.01	0.17±0.01	0.18±0.01
49	2-Heptanone	C110430	$C_7H_{14}O$	114.2	886.9	263.302	1.6276	0.07±0.01	0.09±0.01	0.03±0.01	0.02±0.00	0.04±0.00
60	Cyclohexanone	C108941	$C_6H_{10}O$	98.1	892.9	267.083	1.1582	0.22±0.02	0.06±0.00	0.05±0.00	0.05±0.00	0.05±0.03
55	4-Heptanone	C123193	$C_7H_{14}O$	114.2	866.3	251.076	1.5651	0.09±0.00	0.18±0.02	0.18±0.01	0.21±0.01	0.20±0.01
6	3-hydroxy-2-Butanone	C513860	$C_4H_8O_2$	88.1	700.5	173.979	1.3315	0.70±0.05	1.65±0.04	0.50±0.03	0.47±0.02	1.04±0.09
1	2-Pentanone	C107879	$C_5H_{10}O$	86.1	688.6	35.200	1.3749	1.39±0.04	0.80±0.08	0.72±0.04	0.75±0.04	0.78±0.01
11	2,3-pentandione	C600146	$C_5H_8O_2$	100.1	704.7	175.542	1.2199	0.57±0.03	1.36±0.04	1.76±0.06	1.75±0.12	1.68±0.03
41	Cyclopentanone	C120923	C_5H_8O	84.1	782.9	207.071	1.1057	0.06±0.01	0.04±0.00	0.04±0.00	0.04±0.00	0.06±0.00
73	1-Hydroxy-2-propanone	C116096	$C_3H_6O_2$	74.1	641.4	157.455	1.2243	0.59±0.06	0.99±0.08	1.09±0.13	1.20±0.07	1.13±0.03
7	1-Penten-3-one	C1629589	C_5H_8O	84.1	670.6	164.899	1.3089	1.51±0.36	3.11±0.29	3.35±0.50	3.68±0.39	3.57±0.14
22	2-Propanone	C67641	C_3H_6O	58.1	482.0	122.294	1.1287	8.28±1.28	9.46±0.61	8.74±0.64	8.50±0.40	8.79±0.29
	**Alcohols (9)**											
53	1-Hexanol	C111273	$C_6H_{14}O$	102.2	864.7	250.111	1.6490	0.03±0.00	0.08±0.00	0.08±0.01	0.09±0.00	0.11±0.00
51	(3E)-Hexenol	C928972	$C_6H_{12}O$	100.2	855.4	244.777	1.5296	0.19±0.03	0.29±0.04	0.31±0.02	0.34±0.01	0.30±0.02
54	(Z)-3-hexenol	C928961	$C_6H_{12}O$	100.2	845.1	239.049	1.5124	0.19±0.02	0.23±0.05	0.21±0.04	0.22±0.02	0.18±0.01
75	2-Furanmethanol	C98000	$C_5H_6O_2$	98.1	855.8	245.020	1.3571	0.79±0.07	1.30±0.05	1.30±0.05	1.37±0.06	1.37±0.01
66	2-methyl-butan-1-ol	C137326	$C_5H_{12}O$	88.1	739.7	189.029	1.2308	0.96±0.04	0.48±0.03	0.53±0.05	0.48±0.02	0.48±0.00
70	2-methyl-1-Pentanol	C105306	$C_6H_{14}O$	102.2	834.0	232.976	1.3012	0.68±0.04	0.40±0.02	0.38±0.02	0.37±0.02	0.38±0.01
57	2-Propanol	C67630	C_3H_8O	60.1	479.4	121.772	1.2069	0.57±0.05	0.27±0.04	0.22±0.03	0.17±0.01	0.19±0.02
65	2-Hexanol	C626937	$C_6H_{14}O$	102.2	800.1	215.427	1.2833	2.83±0.14	2.93±0.15	2.91±0.13	3.03±0.10	2.93±0.05

No.	Compound	CAS#	Formula	MW	RI	Rt [sec]	Dt [a.u.]	Average of relative content（%）				
								D0	D7	D14	D21	D28
46	2-Ethoxyethanol	C110805	$C_4H_{10}O_2$	90.1	698.6	173.307	1.0895	0.29±0.03	0.24±0.02	0.16±0.00	0.15±0.01	0.23±0.02
						**Nitrogen oxides (4)**						
24	2-acetyl-3-methylpyrazine	C23787806	$C_7H_8N_2O$	136.2	1083.7	478.687	1.1798	2.93±0.59	2.15±0.10	2.02±0.20	1.95±0.05	1.86±0.03
33	2-Ethyl-5-methylpyrazine	C13360640	$C_7H_{10}N_2$	122.2	993.0	353.317	1.1715	0.57±0.15	0.49±0.01	0.56±0.10	0.44±0.04	0.55±0.03
58	dimethylamine	C124403	C_2H_7N	45.1	405.7	108.338	1.0758	2.01±0.39	9.78±0.39	10.19±0.33	10.30±0.46	10.63±0.28
40	2-Methylpyrazine	C109080	$C_5H_6N_2$	94.1	827.2	229.342	1.1015	0.90±0.12	0.86±0.06	0.98±0.07	0.89±0.02	0.88±0.02
						**Acids (4)**						
56	2-Methylbutanoic acid	C116530	$C_5H_{10}O_2$	102.1	855.7	244.959	1.4725	0.11±0.01	0.17±0.02	0.17±0.02	0.16±0.01	0.16±0.00
45	Acetic acid	C64197	$C_2H_4O_2$	60.1	618.8	151.904	1.0465	1.92±0.13	2.51±0.02	2.40±0.12	2.29±0.21	2.25±0.12
38	Hexanoic acid	C142621	$C_6H_{12}O_2$	116.2	987.1	347.511	1.2996	0.68±0.01	0.47±0.02	0.49±0.02	0.45±0.04	0.41±0.01
42	3-methylbutanoic acid	C503742	$C_5H_{10}O_2$	102.1	867.4	251.716	1.2196	0.38±0.05	0.27±0.02	0.26±0.02	0.27±0.02	0.25±0.01
						**Sulfur-containing compounds (5)**						
62	Propylsulfide	C111477	$C_6H_{14}S$	118.2	881.9	260.302	1.1731	0.26±0.01	0.21±0.03	0.12±0.01	0.08±0.01	0.07±0.00
44	Diethyl sulfide	C352932	$C_4H_{10}S$	90.2	696.7	172.594	1.0448	0.14±0.02	0.12±0.01	0.26±0.01	0.28±0.03	0.19±0.01
37	Furfuryl methyl sulfide	C1438911	C_6H_8OS	128.2	979.0	339.685	1.1418	0.91±0.13	0.42±0.03	0.39±0.00	0.37±0.03	0.37±0.01
34	allyl isothiocyanate	C57067	C_4H_5NS	99.2	880.9	259.646	1.3771	0.74±0.04	0.43±0.05	0.28±0.06	0.20±0.00	0.15±0.01
19	2,4,5-Trimethylthiazole	C13623115	C_6H_9NS	127.2	987.4	347.757	1.5612	3.45±0.40	2.24±0.12	2.16±0.06	2.13±0.07	2.10±0.02
						**Others (8)**						
67	2-ethyl-furan	C3208160	C_6H_8O	96.1	715.9	179.736	1.3059	0.29±0.03	0.69±0.08	0.69±0.03	0.69±0.03	0.60±0.01
47	Benzene	C71432	C_6H_6	78.1	659.7	162.07	1.0878	0.82±0.02	0.49±0.01	0.55±0.08	0.53±0.05	0.53±0.02
23	4-Methylphenol	C106445	C_7H_8O	108.1	1084.1	479.297	1.1429	3.28±0.21	2.36±0.12	2.25±0.15	2.13±0.04	2.04±0.01
15	gamma-Terpinene	C99854	$C_{10}H_{16}$	136.2	1059.8	441.679	1.6899	0.52±0.03	0.89±0.05	0.90±0.15	0.71±0.06	0.78±0.03
16	alpha-Phellandrene	C99832	$C_{10}H_{16}$	136.2	1005.2	367.348	1.6827	3.25±0.41	1.67±0.74	2.30±0.23	2.46±0.39	2.46±0.27
10	2,2-Dimethyl-3-methylenebicyclo[2.2.1]heptane	C79925	$C_{10}H_{16}$	136.2	946.4	310.101	1.7147	0.53±0.03	1.24±0.14	1.39±0.06	1.33±0.11	1.20±0.03
43	1,1-Diethoxyethane	C105577	$C_6H_{14}O_2$	118.2	732.0	185.971	1.0443	0.51±0.02	1.81±0.04	1.87±0.05	1.86±0.15	1.79±0.04

No.	Compound	CAS#	Formula	MW	RI	Rt [sec]	Dt [a.u.]	Average of relative content（%）				
								D0	D7	D14	D21	D28
18	beta-mircene	C123353	$C_{10}H_{16}$	136.2	987.6	347.985	1.6408	0.29±0.01	0.21±0.02	0.18±0.02	0.17±0.01	0.13±0.01
71	1-methyl-2-Pyrrolidinone	C872504	C_5H_9NO	99.1	1048.2	424.749	1.4491	0.15±0.01	0.23±0.01	0.24±0.02	0.23±0.01	0.22±0.03
